# Evidence for holistic episodic recollection via hippocampal pattern completion

**DOI:** 10.1038/ncomms8462

**Published:** 2015-07-02

**Authors:** Aidan J. Horner, James A. Bisby, Daniel Bush, Wen-Jing Lin, Neil Burgess

**Affiliations:** 1 UCL Institute of Cognitive Neuroscience, 17 Queen Square, London WC1N 3AR, UK; 2 UCL Institute of Neurology, Queen Square, London WC1 3BG, UK

## Abstract

Recollection is thought to be the hallmark of episodic memory. Here we provide evidence that the hippocampus binds together the diverse elements forming an event, allowing holistic recollection via pattern completion of all elements. Participants learn complex ‘events' from multiple overlapping pairs of elements, and are tested on all pairwise associations. At encoding, element ‘types' (locations, people and objects/animals) produce activation in distinct neocortical regions, while hippocampal activity predicts memory performance for all within-event pairs. When retrieving a pairwise association, neocortical activity corresponding to all event elements is reinstated, including those incidental to the task. Participant's degree of incidental reinstatement correlates with their hippocampal activity. Our results suggest that event elements, represented in distinct neocortical regions, are bound into coherent ‘event engrams' in the hippocampus that enable episodic recollection—the re-experiencing or holistic retrieval of all aspects of an event—via a process of hippocampal pattern completion and neocortical reinstatement.

## Abstract (web summary)

The holistic retrieval of complex event memories is thought to be the hallmark of episodic memory. Here, the authors provide behavioural and neuroimaging evidence that the hippocampus binds together the elements forming an event to allow holistic episodic recollection via pattern completion of all elements.

## Graphical abstract

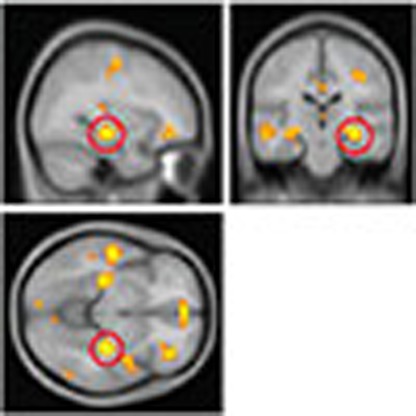

The holistic retrieval of complex event memories is thought to be the hallmark of episodic memory^1^, underpinning the ‘recollective' experience. Episodic recollection is distinct from other forms of memory retrieval, such as retrieval of semantic associations (for example, associating Marilyn Monroe with New York City) or feelings of familiarity. Critically, all aspects of an event are retrieved, including contextual elements that might be incidental to the content of the event, leading to the re-experiencing of a complete multimodal event. A widely held^2^,^3^,^4^,^5^,^6^,^7^ but long-debated^8^ view holds that episodic memory is the key function of the hippocampus, binding together the elements of an event^6^,^9^,^10^, allowing for their retrieval via hippocampal pattern completion^11^,^12^,^13^,^14^,^15^,^16^, and subsequent reinstatement in the neocortex. Despite evidence for pattern separation for simple objects^17^ and neocortical reinstatement of associative information^18^,^19^,^20^,^21^,^22^, evidence for hippocampal pattern completion and holistic reinstatement of all elements for complex events has been lacking.

In line with Marr^11^, we define pattern completion as the retrieval of all constituent elements of an event (that is, the complete pattern) when presented with a single element as a cue (that is, a partial input). If retrieval of a complex event with more than two elements (for example, location, person, object triplets) occurs via pattern completion, two key predictions follow. First, retrievals of different elements from the same event will be statistically related, because the retrieval of any one element depends on the strength of all within-event associations. Second, retrieval of any one element from an event should coincide with reactivation of neurons corresponding to all event elements, including those incidental to the task. To demonstrate how these predictions arise, we show that they can emerge from the canonical attractor network model of hippocampal function^12^.

According to the first prediction, the retrieval of different elements from the same event should be related. For example, an event memory might bind together the location we were in, the person we met and the object they gave us. Later, when cued with the location, our ability to retrieve the person and the object should be related, because retrieving any one type of element involves reactivation of all types of element. That is, episodic recollection should be ‘holistic' in the sense of producing a re-experience of an entire multimodal event^1^. Of course, the encoding and retrieval process will be as error-prone as any other cognitive function: episodic recollection is not generally veridical^23^,^24^, and one can falsely ‘recollect' with associated high confidence^25^. The prediction of holistic recollection via pattern completion is that, due to the presence of a single coherent event engram, all types of elements are reactivated causing performance in remembering the different aspects of the same event to be related (although with a certain degree of error in the retrieval process).

We previously demonstrated the statistical dependency that would be predicted by holistic retrieval, using events created from multiple simultaneously presented elements^26^. We then showed that this dependency likely results from pattern completion at retrieval, rather than fluctuations in encoding strength, because the same statistical dependency can be seen when holistic events are built up from sequentially presented overlapping pairs of elements, so long as all inter-element associations are formed^27^. We could not behaviourally distinguish between the retrieval of ‘events' built across three separate encoding trials and the retrieval of events learnt on a single trial. Thus the key to creating holistically bound event representations lies in the associative structure created between the constituent elements (that is, it is the relations between elements that is critical; see also ref. 28), even without their simultaneous presentation. Here we sought more direct evidence for pattern completion. We show how behavioural dependency results from pattern completion within an attractor network. We then use functional magnetic resonance imaging (fMRI) to provide evidence for the predictions that all event elements are retrieved and reinstated in the neocortex and that this reinstatement is related to pattern completion in the hippocampus.

At encoding, participants learned a series of multi-element ‘events' (Fig. 1). Each event consisted of three or four elements (locations, famous people, objects and animals). Events were built up over three separate encoding trials (interleaved with encoding trials of other events). Each trial consisted of presentation of one of the three possible pairwise associations from an event. This paradigm allows us to build ‘events' with different associative structures of overlapping pairs: ‘closed-loop', in which all event elements were presented paired with all other elements of the event; or ‘open-loop', in which elements of an event were presented as a chain of overlapping pairs. Here, we use the term ‘event' in relation to the set of overlapping associations encoded across separate trials, leaving a consideration of how this relates to an ‘event' in the real world to the discussion. Importantly, within-event dependency occurs for closed-loop but not open-loop associative structures. This allows us to experimentally manipulate the presence or absence of holistic retrieval. Thus, we can compare encoding and retrieval of virtually identical interleaved paired associates, which differ only by whether or not they form part of a closed-loop ‘event', and thus whether or not they should generate pattern completion.

At retrieval, we tested each encoded pairwise association in each event in both directions using cued six-alternative forced choice among elements of the same type from other events (for example, cue location, retrieve the associated person among five people from other events, and cue person, retrieve location). Thus, each event was tested across six separate retrieval trials. Note that a single cue and six target elements were presented on all retrieval trials with the task being to retrieve the paired associate. Thus, as with encoding, the closed-loop and open-loop conditions were exactly matched in terms of stimuli and task demands, differing only in the potential occurrence of different levels of incidental reactivation of associated elements that were irrelevant to the task (that is, different levels of pattern completion).

We assessed dependency by constructing contingency tables for retrieving two elements (for example, person and object) when cued by the remaining element (for example, location), as well as for retrieving one element (for example, location) when cued by the remaining elements (for example, person and object). For each contingency table, we calculated the proportion of events where elements were either both correctly or both incorrectly retrieved, and this measure of dependency was compared with independent and dependent models of retrieval. Each model predicts the level of dependency expected if retrievals of elements from the same event are either independent or dependent, while controlling for factors such as the participant's overall levels of performance and guessing (see Methods and Table 1). Following the MRI session, participants completed a post-scan debriefing where we asked them about the elements they brought to mind during the encoding and retrieval of specific pairwise associations (see Methods).

## Results

### Computational model

Our two predictions relating to the presence of pattern completion were: (1) statistical dependency for retrieval of different associations of the same event and (2) retrieval of all event elements, including those incidental to the task. First, we demonstrate that both predictions can emerge from a canonical computational model of hippocampal function for the closed-loop relative to the open-loop condition. At encoding, closed-loop and open-loop events were formed by separately learning overlapping pairwise associations between neurons coding for individual elements within a fully recurrent attractor network^12^ (see Methods). At retrieval, a single ‘cue' neuron was activated. Six ‘target' neurons of another element type were also partially activated to model the six-alternative forced choice task.

To assess dependency, we converted mean firing rate from the six neurons associated with the six possible alternative ‘targets' on each retrieval trial into a binary ‘correct' or ‘incorrect' response per trial. We then applied the same statistical models to this output as applied to the behavioural data. The model shows the same pattern of dependency as seen in the behavioural data^27^, with a greater difference between the data and the independent model in the closed-loop relative to open-loop condition (Fig. 2).

Finally, the presence of pattern completion predicts the automatic reactivation of neurons corresponding to all event elements. If we cue with the location and retrieve the person, the ‘non-target' object should also be activated. Consistent with this, the mean firing rate for the non-target neuron was higher for closed-loop than open-loop events, confirming the retrieval of all event elements selectively in the closed-loop condition (Fig. 2). Our computational model therefore corroborates our interpretation that the different associative structures of the closed- versus open-loop events can give rise to pattern completion: we see both statistical dependency (as seen in the behavioural data) and retrieval of ‘non-target' elements (as seen below in the fMRI data).

### Behavioural dependency

Mean performance was similar to our previous studies^26^,^27^ (see Supplementary Note 1 for analyses across conditions). As in our computational model, we saw significant behavioural dependency for closed-loop, but not open-loop, events (Fig. 3; see Supplementary Note 2 for analyses of raw dependency across conditions). Dependency was greater than the independent model for closed-loop events (*t*(25)=2.57, *P*<0.05), but not for open-loop events (*t*(25)=1.80, *P*=0.08). Note the trend for open-loop events is in the opposite direction (that is, less dependency in the data than independent model) to that for closed-loop events. Thus, open-loop events did not show behavioural dependency, whereas the closed-loop events did. Importantly, the difference between the data and independent model was greater for closed-loop than open-loop events (*t*(25)=2.77, *P*<0.05), consistent with the presence of pattern completion in the closed-loop condition relative to the open-loop condition. Note, we have previously shown this difference in dependency is not a function of the number of within-event elements, given that no dependency is seen when three elements are encoded via two overlapping associations^27^.

### fMRI results at encoding

As noted in the Methods, we report any non-hippocampal regions that survived *P*<0.05 family-wise error (FWE) correction for the whole brain. Given our specific hypotheses concerning the hippocampus, we used a *P*<0.05 FWE correction within a bilateral hippocampal anatomical mask (that is, a small-volume correction (SVC)).

Using fMRI, we asked where BOLD activity differed between the encoding of different element types (collapsed across closed- and open-loop trials; *P*<0.05 FWE whole-brain corrected). For example, for locations, we compared location–person and location–object/animal with person–object/animal encoding trials. We restricted these element type analyses to the first and second encoding trials, given no location–person associations were encoded on the third pair (that is, we would not have been able to identify regions associated with objects/animals using third pair trials given all trials were either location–object or person–animal trials).

For locations, the greatest difference was seen in bilateral parahippocampal gyrus; for people, the medial parietal and medial prefrontal cortex; and for objects/animals, the lateral occipital and lateral parietal cortex (Fig. 4 and Supplementary Table 1). No differences were seen between objects and animals so a single object/animal region of interest was identified at both encoding and retrieval. Thus, the different types of elements comprising each event produced region specific activity, consistent with the idea that they are represented in distinct neocortical regions.

We next focussed on where the elements in closed-loop events were bound into coherent event memories. We reasoned that activity in such regions during the third encoding trial (that is, the trial which ‘closes the loop') should be predictive of subsequent memory performance (at retrieval) for all the pairwise associations in that event, not just the one being presented. We therefore looked for activity during encoding trials that showed a parametric modulation by subsequent memory performance on the other associations from the same event, and did so more for closed- versus open-loop events. For example, during encoding of a location–person pair, activity would reflect the combined performance across retrieval trials for the location–object/animal and person–object/animal association for that event.

**Figure 1:**
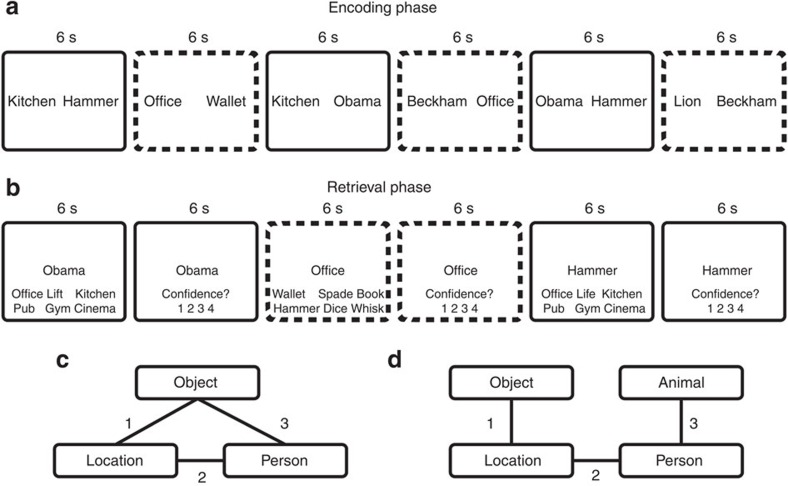
Experimental design (**a**) Encoding phase. Participants saw multiple paired associates. They imagined each pair ‘interacting in a meaningful way as vividly as possible' for 6 s. Each pair was preceded by a 500-ms fixation cross and followed by a 1,500-ms blank screen. Solid lines and dotted lines were not present, but highlight a closed-loop (solid lines) and open-loop (dotted lines) event. The encoding phase was split into three mini-blocks of 36 trials, one mini-block for each of the three pairwise associations for each event, that is, pairs from all the ‘events' were fully interleaved. (**b**) Retrieval phase. Participants were presented with a single cue and required to retrieve one of the other elements from the same event from among five foils (elements of the same type from other events) within 6 s. This was followed by a 1–4 confidence rating within 6 s. Each cued-recognition/confidence judgement trial was preceded by a 500-ms fixation cross and followed by a 1,500-ms blank screen. (**c**) The associative structure of closed-loop events, with example encoding order for the three pairwise associations (numbers 1–3). (**d**) The associative structure of open-loop events, with example encoding order for the three pairwise associations.

This analysis was performed separately for the first, second and third encoding trials of open- and closed-loop events (note that encoding trials for open- and closed-loop events only differed in whether the final third trial ‘closed the loop' or formed an open chain, Fig. 1). This analysis revealed differential activity in bilateral anterior hippocampus for the third encoding trial when comparing closed- versus open-loop events (*P*<0.05 FWE small-volume corrected within bilateral hippocampus; Fig. 4 and Supplementary Table 2). Thus, BOLD activity when encoding the third pair of closed-loop events more closely predicted subsequent memory for the other (previously encoded) associations of that event than for open-loop events, consistent with the idea that the hippocampus binds the multiple elements of an event into a single coherent memory trace, enabling pattern completion at retrieval. Critically, our effect was specific to the third encoding trial of ‘closed-loop' associative structures; no differences were seen between closed-loop and open-loop events in the first and second encoding trials. It is only when the last within-event association (which forms a closed-loop structure) is encoded that the hippocampus binds the elements of an event into a coherent event engram.

### fMRI results at retrieval

At retrieval, we again looked for BOLD activity differences specific to the type of element being cued or retrieved (again, collapsed across closed- and open-loop trials). For example, for locations, we compared trials that cued or retrieved locations versus trials where locations were neither cued nor retrieved (in these trials, locations are referred to as ‘non-targets'). As at encoding, the peak response for locations was in bilateral parahippocampal gyrus, for people it was in medial prefrontal cortex and for objects/animals it was in lateral parietal cortex (*P*<0.05 FWE whole-brain corrected; Fig. 5 and Supplementary Table 3).

### Non-target reinstatement in the neocortex

We next extracted the BOLD responses from these three regions in each retrieval trial and examined how they depended on whether the element associated with that region was a cue, a target or a non-target in that trial. For example, cuing with a person and retrieving an object would be a ‘non-target' trial for location and thus for the parahippocampal gyrus. If holistic retrieval is occurring in the closed-loop condition, the region associated with the non-target element should show higher activity for closed- than open-loop events. In the above example, the parahippocampal gyrus (associated with the non-target location) should show greater activity for closed- than open-loop events. Collapsing across the three regions of interest, we saw greater activity for closed-loop than open-loop events during the non-target trials (*t*(25)=3.40, *P*<0.01), but not for cue or target trials (*t*<0.62, *P*>0.54; Fig. 5). Importantly, this effect did not differ across the three regions of interest (see Supplementary Note 3 and Supplementary Fig. 1). Thus, the difference between closed-loop and open-loop events for non-target trials was consistent across regions. This lack of difference across the three regions is important as it suggests that our results are not affected by the identification of a region coding for both objects and animals.

Finally, we conducted a further analysis to rule out that this non-target reinstatement effect was being driven by a specific subset of retrieval trials in which the object/animal is the cue. In these trials, the cue (for example, object) and non-target (for example, person) have been directly associated in closed-loop events but not in open-loop events, so that the non-target region might be more active in the closed-loop versus open-loop condition due to this direct association from the cue. Importantly, the closed-loop > open-loop non-target effect in these trials did not significantly differ from the other trials where both conditions had a direct association between the cue and non-target (that is, trials in which the cue was a person or a location; see Supplementary Note 4). This further underlines that the key to non-target reinstatement, and therefore pattern completion, is the all-to-all associative structure in the closed-loop relative to the open-loop condition.

Thus, activity is similar in regions associated with the cue and the target between closed-loop and open-loop events, consistent with the presence of simple associative retrieval in both conditions. Critically, regions associated with the non-target element were specifically reactivated during retrievals from closed-loop but not open-loop events, supporting the idea that, for closed-loop events, the representations of all constituent elements are reinstated in the neocortex.

### Non-target reinstatement and hippocampal activity

We have so far provided evidence that, for closed-loop events (but not for open-loop events), the hippocampus is involved in encoding coherent representations, and the neocortical representations of non-target elements are reinstated during retrieval, both consistent with holistic recollection. We next asked whether, during retrieval of closed-loop (but not open-loop) events, neocortical non-target reinstatement was related to activity in the hippocampus, as would be predicted if holistic reinstatement is driven by hippocampal pattern completion. In other words, regions showing activity correlated with the neocortical reinstatement of non-target elements from closed-loop events are likely to be involved in pattern completion.

We therefore looked, across participants, for activity during retrievals from closed- versus open-loop events that correlated with the extent of neocortical reinstatement for the non-target (that is, activity in the region corresponding to the non-target during retrievals from closed- versus open-loop events). This analysis revealed correlated activity in bilateral mid-hippocampus (*P*<0.05 FWE small-volume corrected within bilateral hippocampus; Fig. 5 and Supplementary Table 4). Thus, the size of the non-target cortical reinstatement effect correlated with the difference in BOLD response for closed- versus open-loop events in the hippocampus.

Importantly, this correlation does not appear to be driven by the increased hit rate seen for closed-loop relative to open-loop events. The same bilateral hippocampal effect is seen (albeit at a reduced *P*<0.001 uncorrected threshold) when correlating neocortical reinstatement with the difference between closed-loop and open-loop hits (that is, equating memory performance across the conditions by excluding misses). Finally, no correlation is seen in the hippocampus between reinstatement and the difference between hits and misses (collapsed across closed-loop and open-loop events). Thus, the hippocampal effect is specific to the closed-loop condition, and is driven by pattern completion as opposed to overall memory performance.

As an independent check of this effect, we examined the simple contrast of retrievals from closed- versus open-loop events. This shows activity in the right mid-hippocampus at a lenient threshold (*P*<0.005 uncorrected) that overlaps with the activity in the correlation analysis, consistent with hippocampal support of the greater overall neocortical reinstatement for closed- versus open-loop events, albeit via a less sensitive analysis. Reassuringly, the activity in this contrast (that is, mean activity in the right mid-hippocampal region of interest) per participant correlates with that participant's neocortical reinstatement activity, as would be expected from the whole brain correlation analysis (Fig. 5). Overall, these results provide evidence that hippocampal activity at retrieval is related to the reinstatement of all elements of an event, consistent with its hypothesized role in pattern completion.

## Discussion

The defining characteristic of episodic memory is thought to be holistic recollection—the subjective re-experiencing of a complete multimodal event (whether or not its constituent elements are veridical)^1^. Here, we provided fMRI, behavioural and computational modelling evidence for a mechanistic account of episodic recollection based on hippocampal pattern completion and neocortical reinstatement^11^,^12^,^13^,^14^,^15^,^16^. We showed the constituent elements of complex events are represented in distinct neocortical regions, but bound into a coherent ‘event engram' in the hippocampus. At retrieval, a partial cue (that is, a single element) can result in the retrieval of all event elements, not just the target of retrieval, via hippocampal pattern completion, and their reinstatement in the neocortex.

At encoding, hippocampal activity was predictive of subsequent memory. Although evidence has been provided for a correlation between BOLD response on individual trials and subsequent memory for the items encoded on that trial^29^,^30^, here we provide evidence for a correlation between BOLD response when learning one pair (for example, a B–C pair) and performance on the other overlapping pairs from the same event (that is, the A–B and A–C pairs). One study has shown BOLD activity during learning of A–C pairs predicts performance for previously learned A–B pairs^31^. However, our effect was only seen in the third encoding trial, and specifically in the closed-loop condition. Thus, it is only when participants ‘close the loop' that the hippocampus binds all the elements of an event into a single coherent ‘event engram'. We therefore provide evidence that the hippocampus is involved in the encoding of complex event engrams that enable holistic recollection via pattern completion, over and above its well-known involvement in the encoding of simple pairwise associations.

Previous studies have also focussed on the extent to which reactivation at encoding^32^ or post-encoding^33^,^34^ predicts subsequent memory performance and generalization to new information. For example, when learning A–B and then A–C pairs, the reactivation of element ‘B' when learning the A–C pair predicts performance when participants are later tested on non-encoded B–C pairs^32^. Such reactivation might form a weak association between B–C pairs, allowing participants to generalize at retrieval. We saw this in our previous study, where participants were above chance performance when retrieving non-encoded B–C pairs despite not showing behavioural dependency (that is, pattern completion) for these open-loop associative structures^27^. However, explicit (as in the present study) or repeated^35^ learning of such associations might be necessary to form strong enough associations between all constituent elements to allow conscious holistic retrieval (see ref. 27 for further discussion). Put another way, learning of A–B/A–C associative structures might still allow for pattern completion (for instances in which a B–C association is formed), however, the probability of this occurring will be less than in our ‘closed-loop' condition.

Theoretical models of the hippocampus suggest that memory traces are retrieved via pattern completion^11^,^12^,^14^,^16^, so that a partial input can result in the retrieval of the complete memory. In particular, region CA3, with its dense recurrent collaterals, is thought to support pattern completion. Consistent with these models, and with evidence for recollection-like processes in rodents^36^, the firing patterns of place cells in the rodent hippocampus switch abruptly when the environment is morphed between two familiar configurations^37^, and the robustness of place cell firing to removal of subsets of environmental cues is disrupted in mutant mice with disabled NMDA receptors in CA3 (ref. 13). However, evidence for pattern completion in the human hippocampus for complex events has been lacking, in contrast to recent findings regarding pattern separation for simple objects^17^. Our results suggest that these models can be extended to the human hippocampus, and that the same underlying mechanisms allow for retrieval of memory traces in the hippocampus across species. Further, we show that the canonical model of hippocampal pattern completion applies to complex multi-element events, thought to be the fundamental unit of episodic memory^1^.

Evidence exists for reinstatement (at retrieval) of coarse stimulus categories^18^,^19^,^20^,^38^, low-level visual properties^39^, encoding task context^21^ and trial unique associative information^40^. These studies have focussed on the element the participant is explicitly required to retrieve (though see also refs 41, 42). Here, we extend them to provide evidence for reinstatement of event elements that are incidental to the retrieval task. This incidental reinstatement is a direct predicted consequence of pattern completion in attractor network models of memory (as seen in our computational model). We cue with one element and the participant is explicitly required to retrieve a second element of the event. The third event element is neither the cue nor the retrieval target. Nonetheless, we see the consequences of its retrieval in the neocortex consistent with the presence of pattern completion. For example, when cued with a location and asked to retrieve a person, there was no explicit requirement to retrieve the object. Despite this, we saw reinstatement of the object, consistent with all elements of a fully bound (closed-loop) event being retrieved by pattern completion and reinstated in the neocortex. These findings are consistent with the observation that retrieval of an object also results in the incidental reactivation of the neural representation of the location associated with the object^43^, linking these observations to retrieval of complex multi-element events. Finally, the increased within-event dependency for closed-loop ‘events' was related to participants' subjective experience. A post-scan debrief revealed that participants were more likely to ‘bring to mind' the non-target element for closed-loop than open-loop events, and that this non-target retrieval correlated with the level of behavioural dependency across participants (*R*^2^=0.19, *P*<0.05; Fig. 3; see Supplementary Note 5 for in-depth analyses of post-scan debrief data).

We also provided evidence that hippocampal activity correlates with the extent of incidental neocortical reactivation. This finding complements fMRI evidence for a trial-wise correlation between hippocampal activity and neocortical reinstatement^22^, as well as connectivity analyses showing greater hippocampal–neocortical connectivity during successful retrieval of pairwise object–scene associations^44^. However, here we show a specific relationship between hippocampal activity and incidental reactivation in the closed-loop condition. In other words, the hippocampal–neocortical relationship is consistent with the presence of hippocampal pattern completion and holistic neocortical reinstatement. Finally, recent evidence has shown the hippocampus to be critically involved in neocortical reinstatement in contextual fear conditioning in mice^45^. Thus, our results provide a link from animal models of hippocampal–neocortical interaction, suggesting the same neural mechanisms underpin our ability to retrieve and re-experience complex episodic events.

Our separated encoding presentation allowed us to study pattern completion resulting from an all-to-all associative structure that would normally be formed by simultaneous presentation, while controlling for temporal variations in encoding strength. In short, across encoding trials, we built associative structures similar in nature to those that would be formed for a typical (simultaneously experienced) ‘event', with similar levels of within-event dependency at retrieval^27^. The critical point here is that although the associative structures in our experiment are not formed in a typical real-life ‘event'—that is, a single spatiotemporal instance—our behavioural findings suggest that a similarly holistic engram of the event is formed in the closed-loop condition^27^. Our results suggest that it is the all-to-all associative structure that is critical, automatically leading to pattern completion and the retrieval of all associated event elements. Thus, the hippocampus may support episodic memory via a more general role in associative/relational encoding and retrieval, whether implicit or explicit^6^,^46^,^47^,^48^, which might also support the integration of recently presented information^32^,^49^ and its binding with related experience^50^.

Importantly, we compare the presence of pattern completion in the closed-loop condition to similarly complex ‘open-loop' associative structures. In the case of open-loop events, we did not find evidence for pattern completion, seeing neither behavioural dependency nor non-target reinstatement for these events. Critically, it is by comparing the closed-loop to open-loop condition that we are able to provide evidence for pattern completion, over and above simple pairwise associative retrieval (for example, as might be expected within semantic memory). The latter is occurring in both conditions whereas the former is only present for closed-loop events—as indexed by both behavioural dependency and non-target neocortical reinstatement.

**Figure 2:**
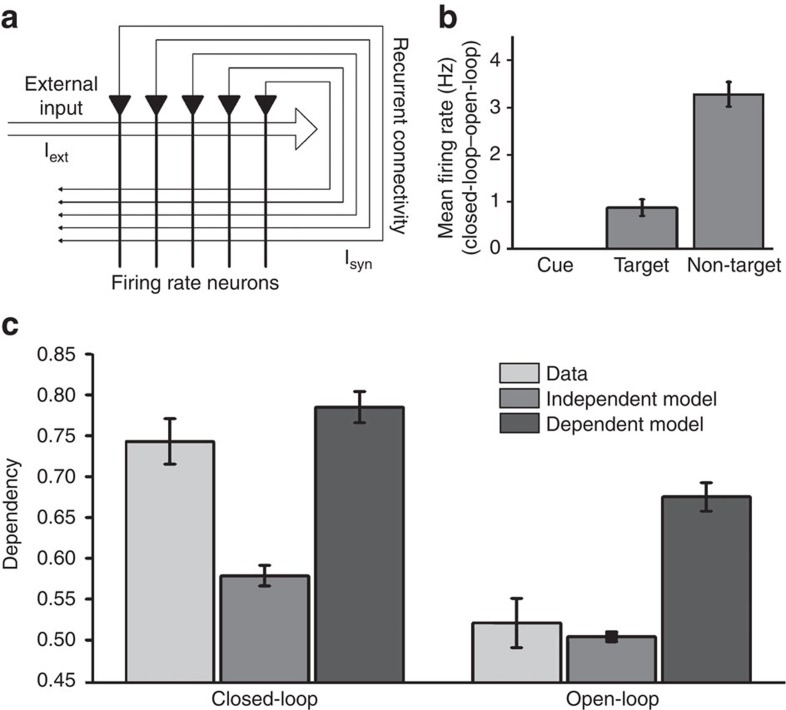
Computational model (**a**) Schematic of fully recurrent attractor network model, in which each element is represented by a unique neuron and synaptic connections show Hebbian plasticity. (**b**) The difference in the mean firing rates of neurons representing the cue, target and non-target between closed-loop and open-loop events. Note, the mean and standard error for the difference cue activity are too small to be shown at this *y* axis scale. (**c**) Predicted dependency from the model between multiple retrievals of different associations from the same event, for closed-loop and open-loop events, and corresponding independent and dependent models. See text for details, including the mapping between firing rates and performance. Error bars±1 s.e. The error bars represent the standard error across 10 differently seeded simulations.

**Figure 3:**
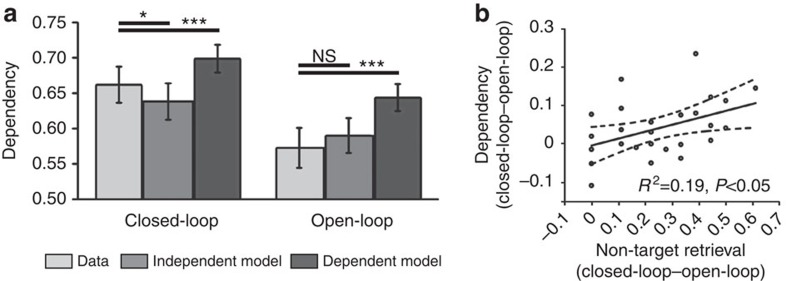
Behavioural results (**a**) Behavioural dependency between multiple retrievals from the same event, for closed-loop and open-loop events and corresponding independent and dependent models. Error bars±1 s.e. ***P<0.001; *P<0.05; NS, not significant. (**b**) Across-participant correlation between behavioural dependency (closed- versus open-loop events, relative to independent model baseline) and how often participants ‘brought to mind' non-target elements at retrieval (that is, the element not cued or retrieved), assessed in the post-scan debrief session (proportion of events where non-target element retrieved for closed-loop versus open-loop events). *N*=26 for both **a** and **b**.

**Figure 4:**
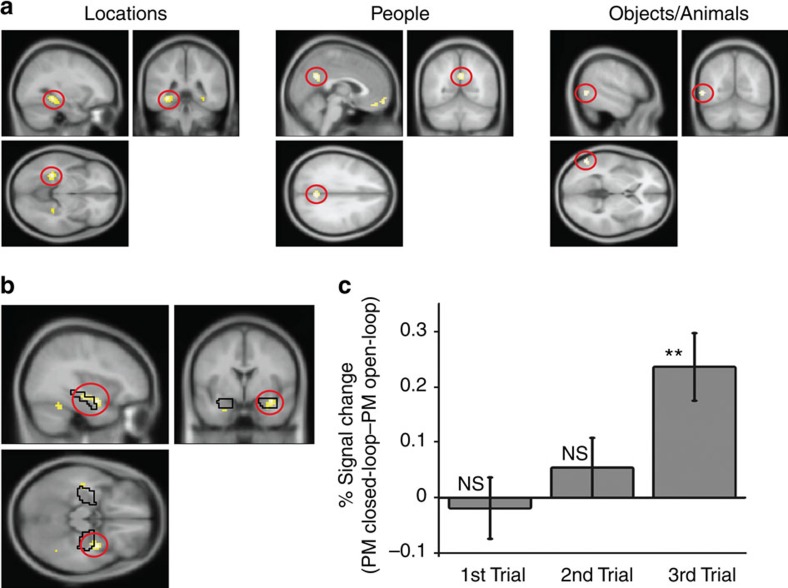
Encoding of events (**a**) Cortical regions showing greater activation at encoding for locations (parahippocampal gyrus; MNI coordinates: −30 −40 −8), people (medial parietal cortex; +3 −58 +31) and objects/animals (lateral occipital cortex; −54 −64 +1). Each contrast is relative to encoding trials that do not include the element of interest. (**b**) Regions showing a greater subsequent memory effect for retrieval of the other paired associates from that event in the closed-loop than open-loop condition during the third encoding trial. The medial temporal lobe cluster includes the right anterior hippocampus (+36 −13 −14). The black outline shows the hippocampal region used for the small-volume correction, with the cluster extending into this volume. (**c**) Mean % signal change for the subsequent memory parametric modulator (PM; across the entire range of values 0–4) in a 5-mm sphere centred on the peak hippocampal voxel from **b** across encoding trials (closed-loop versus open-loop events). Error bars±1 s.e. Activations in **b** shown at *P*<0.001 uncorrected (cluster size >20 voxels) across the whole brain (hippocampal region survives *P*<0.05 FWE SVC), all other activations shown at *P*<0.05 FWE corrected across the whole brain, see Methods. **P<0.01, NS, not significant. *N*=26 for all the plots.

**Figure 5:**
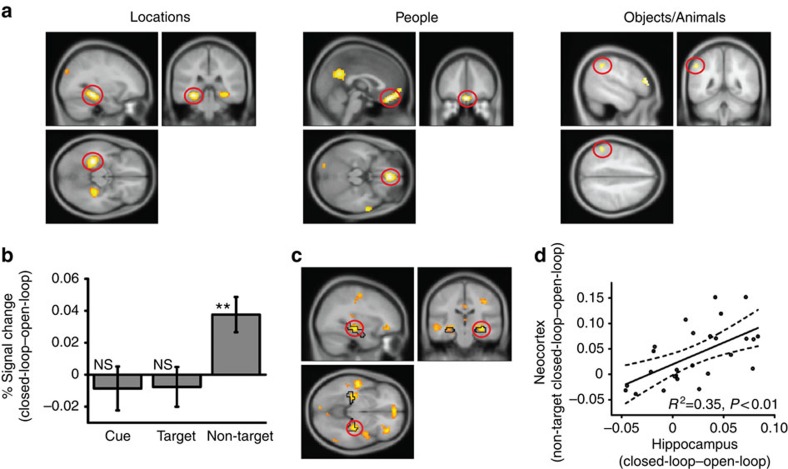
Retrieval of events (**a**) Cortical regions showing greater activation at retrieval for locations (parahippocampal gyrus; −30 −34 −14), people (medial prefrontal cortex; 0 +41 −20) and objects/animals (lateral parietal; −51 −46 +43). Each contrast relates to the element of interest being a cue or target relative to a non-target. For example, for locations, we contrasted location–person and location–object/animal pairs with person–object/animal pairs. (**b**) Mean % signal change for the cue, target and non-target (closed-loop versus open-loop events; mean across the three element-specific regions of interest). Error bars±1 s.e. (**c**) Regions showing a correlation between activity during retrievals from closed-loop versus open-loop events and activity in the region representing the non-target element during retrievals from closed-loop versus open-loop events, showing bilateral mid-hippocampus and highlighting the peak correlation in the right hippocampus (+33 −22 −8). The black outline shows the hippocampal region used for the small-volume correction, with the cluster peaking in this volume. (**d**) Correlation between the difference in hippocampal activity during retrievals from closed-loop versus open-loop events (right mid-hippocampal ROI defined by the main closed-loop versus open-loop contrast, +30 −31 −5) and activity in neocortical regions representing the non-target element at retrieval (closed-loop versus open-loop events). Activations in **c** shown at *P*<0.001 uncorrected (cluster size >20 voxels) across the whole brain (hippocampal region survives *P*<0.05 FWE SVC), all other activations shown at *P*<0.05 FWE corrected across the whole brain, see Methods. *N*=26 for all the plots.

Pattern completion arises from the different associative structure of the closed-loop versus open-loop events in our study. For closed-loop events, the non-target element can be retrieved via two routes. If cuing with location (L) and retrieving person (P), the object (O) can be retrieved via the L–O association and the L–P/P–O associations. For open-loop events, the object can only be retrieved via the L–O association. This interpretation is corroborated by simulations of the canonical attractor network model (Fig. 2), in which the difference in associative structure leads to pattern completion in the closed-loop relative to the open-loop condition. Thus, the all-to-all recurrent associative structure of our ‘closed-loop' events (formed from six associations between three elements) may be a boundary condition for the occurrence of pattern completion, which was not seen for our ‘open-loop' events (formed from six associations between four elements). Previous work showed that behavioural dependency was also seen for simultaneous presentation of three elements, but not for sequential presentation of two associations between three elements^27^. Further work is needed to reveal the boundary conditions for pattern completion to occur in other associative structures, and whether it can be generated for open-loop structures under different experimental conditions.

In summary, our results provide new evidence supporting the conception of episodic memory by Tulving^1^, and that the hippocampus is specifically involved in the proposed mechanism of episodic recollection^2^,^3^,^4^,^5^,^6^,^7^ over and above its more general involvement in explicit retrieval of pairwise associative information^8^. Our results provide the first support for a specific mechanism of episodic recollection^11^,^12^,^14^,^16^ in which hippocampal pattern completion allows for holistic neocortical reinstatement and thus the re-experiencing of entire complex multi-element events.

## Methods

### Participants

In all, 26 participants (11 female) were recruited through the UCL Institute of Cognitive Neuroscience subject panel (sample size consistent with prior behavioural experiments^27^). All the participants gave informed consent and were reimbursed for their time (£20). They had a mean age of 22.5 years (s.d.=3.6). By self-report, all the participants were right-handed and free from neurological impairment. The experiment was approved by the University College London Research Ethics Committee (1825/003).

### Materials

Stimuli were 36 locations (for example, a kitchen), famous people (for example, Barack Obama), objects (for example, hammer) and animals (for example, dog). Four randomized sets of ‘events' (that is, location–person–object–animal quadruplets) were created and rotated across participants. For each participant, half the four-element sets were randomly assigned to the closed-loop condition and half to the open-loop condition. For the closed-loop condition, half the four-item sets were assigned to be location–person–object events and the other half location–person–animal events. For the open-loop condition, all events used all four elements. This resulted in 18 closed-loop events (nine location–person–object and nine location–person–animal events) and 18 open-loop events.

### Procedure

During the encoding phase, each ‘event' was encoded across three separate trials, with each trial presenting one of the possible pairwise associations for a specific event (total trial number=108). The encoding phase was split into three mini-blocks. One pairwise association for each of the 36 events was presented during each mini-block. Presentation order within each mini-block was randomized. For the closed-loop events, the order of presentation across the mini-blocks for a specific event was either: (1) location–object/animal, location–person and person–object/animal, (2) person–object/animal, location–person and location–object/animal, (3) location–person, location–object/animal, person–object/animal or (4) location–person, person–object/animal and location–object/animal. For open-loop events, it was either: (1) location–object, location–person, person–animal, (2) person–animal, location–person, location–object, (3) location–person, location–object, person–animal or (4) location–person, person–animal, location–object. Thus, for the first and second mini-blocks, the closed-loop and open-loop conditions were identical, presenting a single pairwise association and then a second overlapping pairwise association. The third encoding trial either formed an all-to-all associative structure in the closed-loop condition or an associative chain in the open-loop condition (Fig. 1).

Each encoding trial started with a 500-ms fixation cross, followed by the pairwise association being presented as words to the left and right of fixation for 6 s. During this time, participants were required to ‘imagine the two elements interacting in a meaningful way as vividly as possible'. Each encoding trial finished with a 1,500-ms blank screen. There was no response requirement during encoding.

At retrieval, each event was tested across six separate retrieval trials (total trial number=216). The retrieval phase was split into six mini-blocks, with each event tested once per mini-block. The order of presentation within a mini-block was randomized. The testing order, across mini-blocks, for each event was also randomized. Each pairwise association of each event was tested in both directions (for example, cue location, retrieve person and cue person, retrieve location). For a single retrieval trial a cue (for example, location) was presented at fixation. The target (for example, person) was presented below fixation with five other foils of the same element type (that is, people from other events, randomly selected from all events regardless of closed- or open-loop status). Participants were required to select the element originally paired with the cue element from among the six alternatives presented. Following this, they were required to make a 1–4 confidence rating.

Each retrieval trial started with a 500-ms fixation cross. The cue and six-alternatives were then presented until a response was made (up to a maximum time of 6 s). Following this, the target and five foils were removed from the screen, with the cue remaining for 500 ms. The 1–4 confidence scale was then presented at the bottom of the screen until a response was made (up to a maximum time of 6 s). The trial finished with a 1,500-ms blank screen.

Note, closed- and open-loop events contain equal numbers of overlapping pairwise associations, so differences between them cannot be explained by this factor. The open-loop events are constructed of four elements, compared with three elements in the closed-loop events. Although this difference could potentially lead to differences in hippocampal activity, they would likely be in the opposite direction to that predicted here (that is, greater activity associated with open-loop events comprising more elements). Thus, any difference seen between the two types of event is likely a result of their associative structure and consequent pattern completion, as opposed to the number of pairwise associations or number of elements in each event.

### Post-scan debrief

Following the main fMRI experiment, all participants took part in a debrief session lasting ∼30 min. They were verbally presented with the last encoded pair from each event (36 pairs) and asked a series of questions for each pair. Their answers were given verbally and recorded by the experimenter. For each pair, they were asked nine questions: (1) do you remember seeing this pair in the experiment (either at encoding or retrieval), (2) do you remember seeing this pair at encoding, (3) if so, how did you imagine them interacting and (4) how vividly did you image the pair interacting (on a scale of 1–4), (5) did anything else come to mind when you were imagining the items interacting and (6) if so, did you bring any other elements to mind? This was followed by (7) do you remember being tested on this pair of items, (8) did anything else come to mind when being tested on these items and (9) did you bring any elements to mind?

### Behavioural analyses

Each event was tested across six retrieval trials, resulting in 108 retrieval trials. We first calculated performance across these trials. Performance was analysed in relation to closed-loop versus open-loop events as well as in relation to the cue-type (location, person and object/animal) and target-type (location, person, object/animal).

To assess dependency, we first constructed contingency tables for each participant for (1) retrieving two elements of an event across separate trials when cued by the other element of the event (the AbAc analysis) and (2) retrieving a single event element across separate trials when cued by the other two event elements. Each table shows how performance retrieving one association from an event depends on performance retrieving another (overlapping) association from that event. Each analysis was done in relation to (1) the location being cued—the location AbAc analysis, (2) the location being retrieved—the location BaCa analysis, (3) the person being cued—the person AbAc analysis and (4) the person being retrieved—the person BaCa analysis. Thus, each contingency table was always related to a common (cue or target) element and assessed performance for two overlapping associations (for example, location–person and location–object).

For each contingency table, we created an independent and dependent model. These models were used to estimate the amount of behavioural dependency for a specific participant given various factors such as their overall performance and level of guessing. The independent model estimates the level of dependency expected if the retrieval of elements within an event are independent. This is calculated by multiplying the probabilities of separately retrieving two elements. For example, if assessing dependency for retrieving the person and object when cued by location, for the ‘correct-correct' cell of the table we multiply the probability of retrieving the person when cued by the location across all (*N*) events (*P*_AB_) with the probability of retrieving the object when cued by the location across all events (*P*_AC_), see Table 1 and ref. 27.

The dependent model builds upon the independent model by introducing an ‘episodic factor' (*E*^*i*^) that weights performance for event *i* by the extent to which performance for that event across multiple retrieval trials differs from performance across all events. For example, when retrieving B when cued by A for event *i*:


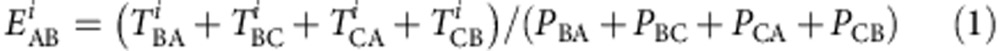


where, *T*^*i*^_BA_=1 if the participant correctly retrieves A when cued by B for event *i* (otherwise, *T*^*i*^_BA_=0), and similarly for *T*^*i*^_BC_ and so on. The probability of correctly retrieving an association from event *i* is weighted by the episodic factor for that event, that is, *P*_AB_ becomes *P′*^*i*^_AB_*=E*^*i*^_AB_*P*_AB_. The dependent model also controls for the level of guessing, so that *E*^*i*^ weights the probability of intentional correct retrieval but not the probability of guessing correctly (which is by definition independent). So the dependent model follows the independent model, with *P*^*i*^_AB_ (and similarly *P*^*i*^_AC_ and so on.) replaced by:


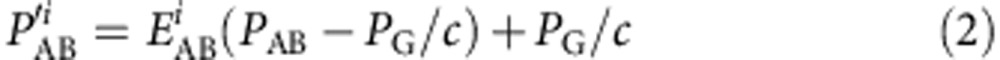


where *P*_G_ is the proportion of guesses, of which *P*_G_/*c* will be correct in *c*-way forced choice cued-recognition (*c*=6 in our experiments). *P*_G_ is estimated as *c*/(*c*−1) times the proportion of errors. The independent model corresponds to setting *E*^*i*^=1 across all the events.

Thus, for each participant, we build contingency tables for the data, independent and dependent models across four analyses (analysis-type—AbAc versus BaCa—and element-type—location versus person). For each table, we calculate a measure of dependency based on the proportion of events where both associations are retrieved correctly or incorrectly, where 1=full dependence and 0.5=full independence. Note, the dependency measure scales with accuracy (and level of guessing), so only comparisons between the data and the models are meaningful. All statistical comparisons are made with two-tailed paired *t*-tests.

### fMRI acquisition

In all, 48 T2*-weighted slices (64 × 74, 3 mm × 3 mm, TR=70 ms, TE=30 ms, repetition time=3,360 ms) per volume were acquired using echo-planar imaging (EPI) on a 3T Trio system (Siemens, Germany) with a 32-channel head coil. Slices were tilted 45° up at the front and acquired in an ascending order. A total 270 volumes were acquired during the encoding phase. The retrieval phase varied in length across participants given each memory judgement was self-paced (up to a maximum of 6 s). The mean number of volumes acquired during retrieval across participants was 478 (range 335–589). The first five volumes of each session (encoding and retrieval) were discarded to allow for T1 equilibrium. A double-echo FLASH field-map for distortion correction of the EPI volumes was acquired, as well as a three-dimensional MDEFT structural image (1 mm^3^) for normalization to a Montreal Neurological Institute (MNI) template image.

### fMRI analyses

*Preprocessing*. Image processing and analyses were performed using SPM8 (www.fil.ion.ucl.uk/spm). The EPI images were first bias corrected to control for within-volume signal intensity differences, unwarped and realigned to correct for movement and slice-time corrected. Each image was then spatially normalized to the MNI template using parameters estimated from warping each participant's structural image to a T1-weighted average template image. Finally, all EPI images were spatially smoothed using an 8 mm FWHM Gaussian kernel.

*General analysis approach*. All statistical analyses were performed in two-stages. In the first stage, neural activity was modelled by either a delta function or boxcar at stimulus onset (dependent on the specific first-level model). The predicted neural activity was then convolved with a canonical hemodynamic response function and down-sampled at the midpoint of each scan to produce regressors for each condition of interest within a general linear model (GLM). Along with the main regressors of interest, all first-level models included six regressors representing the movement parameters estimated during realignment. Parameter estimates for each regressor (condition) were then entered into second-level GLMs to search for consistent effects across participants. All second-level GLMs explicitly modelled subject effects. Unless stated, all effects reported outside the hippocampus are *P*<0.05 FWE corrected. Given our a priori hypotheses regarding hippocampal involvement in the closed-loop condition, we performed SVC (*P*<0.05 FWE) within bilateral hippocampus. The bilateral hippocampal mask was created using the WFU PickAtlas toolbox, with hippocampal regions defined from the Automated Anatomical Labelling atlas. This allows us to identify effects in the hippocampus regardless of location (for example, anterior versus posterior) while still appropriately controlling for multiple comparisons within this volume.

Figures showing SVC results in our hippocampal region of interest are presented at an unmasked *P*<0.001 uncorrected threshold (cluster size >20 voxels) with the outline of the hippocampal region of interest shown in black. Note, we do not formally report any clusters outside the hippocampus that do not survive whole-brain correction as we had no a priori predictions with regards to these regions.

### Encoding phase—element-type

The first-level model consisted of 19 regressors of interest. Separate regressors were made for each of the three mini-blocks. The first and second mini-blocks were associated with six regressors each, one for each possible pairwise association—location–person, location–object/animal, person–object/animal—across the closed-loop and open-loop conditions. Note, further non-reported analyses revealed no significant differences between objects and animals so we collapsed across these categories for all the analyses reported. The third mini-block was associated with four regressors—location–object/animal and person–object/animal pairs across the closed-loop and open-loop conditions (location–person pairs were always encoded on the first or second trial, see above, and so were not present in the third mini-block). We modelled a further three regressors for the mini-block specific inter-trial interval (ITI) period. For each regressor, a boxcar function was used to model the 6-s encoding period or 1.5-s ITI period. Contrasts for all 19 conditions of interest were entered into the second-level analysis.

### Encoding phase—subsequent memory effect

Here, we asked whether the BOLD response on a specific encoding trial was predictive of memory performance for the other associations of that event. For example, if learning a location–person association, the value for the modulator on that trial would be the sum of performance on the four retrieval trials assessing memory for the location–object/animal and person–object/animal association for that specific event. Values therefore ranged from 0 to 4. Separate regressors were created for each of the three mini-blocks across closed-loop and open-loop events. This resulted in 15 regressors; four per mini-block, two relating to the main closed-loop and open-loop regressors and two relating to the parametric modulator for each condition. The final three regressors modelled mini-block specific ITI periods (without any parametric modulators). For each regressor, a boxcar function was used to model the 6-s encoding period or 1.5-s ITI period. The second-level analysis entered contrasts for the six parametric modulators (three mini-blocks × closed-loop versus open-loop) to search for differences between the modulators across conditions.

### Retrieval phase—element-type

Similar to encoding, we searched for BOLD level changes dependent on element-type. At the first-level, 13 regressors were modelled. These related to each cue-target pairing—location–person, person–location, location–object/animal, object/animal–location, person–object/animal, object/animal–person—across the closed-loop and open-loop conditions. The final regressor was the ITI period. For each regressor relating to a retrieval trial, a boxcar function modelled the time from cue/target onset to the time a response was made by the participant (up to a maximum of 6 s). The ITI was modelled with a boxcar function lasting 1.5 s. Contrasts for all the 13 regressors were included in the second-level analysis.

At the second level, we identified cortical regions that showed greater BOLD response to cuing/retrieving each element-type. For example, for locations, we contrasted trials where the location was the cue or target versus trials where the location was the non-target (collapsed across closed-loop and open-loop conditions). This revealed three element-specific regions of interest (ROIs; see Results), from which we extracted the BOLD response for each individual for each of the 13 conditions. For each region, we then calculated the BOLD response when the element associated with that region was the cue, the target and the non-target, split by closed-loop and open-loop conditions. For example, for the parahippocampal region associated with location, we calculated the BOLD response for trials where a location was a cue, a target and a non-target. Thus, for each of the three ROIs, we obtained an estimate of the BOLD response across six conditions—cue, target and non-target × closed-loops versus open-loops.

### Retrieval phase—closed-loop versus open-loop

This model included three regressors, retrieval trials for the closed-loop and open-loop conditions and the ITI. Each regressor relating to a retrieval trial was a boxcar function modelling the time from cue/target onset to the time a response was made by the participant (up to a maximum of 6 s). The ITI was modelled with a boxcar function lasting 1.5 s. Contrasts for all three regressors were included in the second-level analysis.

### Retrieval phase—correlating ‘cortical reinstatement' for non-targets

Here, we were interested in correlating (across participants) the cortical ‘reinstatement' effect with the main closed-loop versus open-loop contrast across the whole brain. The data for the second-level model was the closed-loop versus open-loop contrast from the ‘Retrieval phase—closed-loop versus open-loop' analysis. The GLM included one main regressor (and a second regressor for the intercept) that was the difference in ‘cortical reinstatement' for the non-target element between the closed-loop and open-loop condition. This was calculated by taking the mean difference between closed-loop and open-loop conditions, specifically for the non-target condition, across the three ROIs identified in the ‘Retrieval phase—element-type' analysis. Given this GLM was focussing on differences across participants, no subject effects were included.

### Computational model

We simulated a simple network of *N* rate-coded neurons (equation 3) that were fully recurrently connected except for self-connections (Fig. 2). The firing rate *r*_*i*_ of these neurons was dictated by a time constant *τ*_*r*_=25 ms, a combination of externally applied currents *I*_*i,*ext_ and recurrent synaptic currents *I*_*i*,syn_, and a sigmoidal transfer function (equation 4). We parameterized the transfer function with a threshold *r*_*t*_=10 and a peak firing rate of *r*_max_=10 Hz. All firing rates *r*_*i*_ and synaptic connections *w*_*ij*_ within the network were initially set to zero.


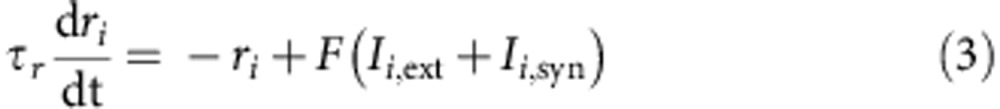



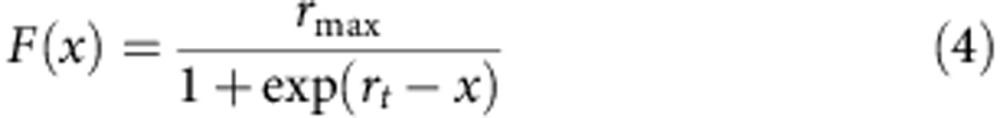


Each element of an event was represented by a unique neuron. During encoding, neurons that represented the stimuli being presented in each trial were externally stimulated with a constant current of *I*_ext_=15 for a period of *t*_enc_=1,000 ms. During this period, synaptic weights developed according to a standard Hebbian learning rule, that is, proportional to the product of pre- and postsynaptic firing rates and learning rate *k* (equation 5). Recurrent synaptic currents were set to *I*_syn_=0 to prevent interference between encoding and retrieval processes. Importantly, to model variation in (extracellular and intracellular) conditions across encoding trials, the learning rate varied from trial to trial, being sampled from a Gaussian distribution with mean *μ*_*k*_=0.55 × 10^−5^ and standard deviation *σ*_*k*_=0.4 × 10^−5^, with negative values set to zero. The encoding order and resulting associative structures for the closed-loop and open-loop condition were identical to the main fMRI experiment.


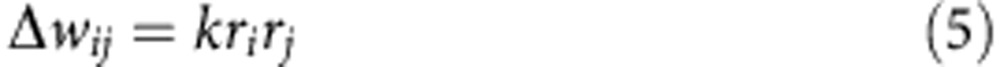


During retrieval, the neuron that represented the cued element received a constant current *I*_ext_=15 for a period of *t*_ret_=1,000 ms, while neurons that represented the six forced choice target elements received a constant current of *I*_ext_=6. Additional activity was generated by the recurrent synaptic current *I*_syn_, which is the product of the synaptic weights and firing rates of connected neurons (equation 6). The learning rate was set to *k*=0 to prevent further encoding.


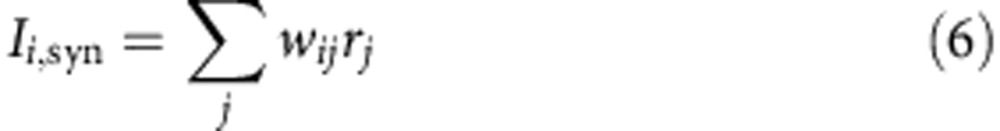


To convert firing rates in a retrieval trial into performance on that trial, the mean firing rate of the neuron representing the target element was expressed as a proportion of the net firing rate of all six neurons and compared with a threshold to determine whether the response was correct or incorrect. This threshold was set to the 36th percentile of the proportional target element firing rate measure across all retrieval trials (regardless of the closed-loop/open-loop condition) so as to match behavioural performance in the main experiment (64%). Statistical dependency was then computed as described above in relation to the behavioural data. The retrieval order for each pairwise association for the closed-loop and open-loop conditions was identical to the main experiment. Ten simulations (with different seeds for encoding variation) were performed, each containing 36 events (18 closed-loop and 18 open-loop).

## Additional information

**How to cite this article:** Horner, A.J. *et al.* Evidence for holistic episodic recollection via hippocampal pattern completion. *Nat. Commun.* 6:7462 doi: 10.1038/ncomms8462 (2015).

## Supplementary Material

Supplementary Information Supplementary Figure 1, Supplementary Tables 1-4 and Supplementary Notes 1-5

## Figures and Tables

**Table 1:** Contingency tables for the independent and dependent models, giving the frequency (over events) of the four combinations of correct or incorrect retrieval of elements B and C when cued by element A.

**Retrieval of element (C)**	**Retrieval of element (B)**	
	**Correct (*****P***_**AB**_)	**Incorrect (1−*****P***_**AB**_)
*Independent model*		
Correct (*P*_AC_)	∑_*i*=1_^*N*^ *P*_AB_*P*_AC_	∑_*i*=1_^*N*^ *P*_AC_ (1*−P*_AB_)
Incorrect (1−*P*_AC_)	∑_*i*=1_^*N*^ *P*_AB_ (1*−P*_AC_)	∑_*i*=1_^*N*^ (1*−P*_AB_)(1*−P*_AC_)
		
*Dependent model*		
Correct (*P*_AC_)	∑_*i*=1_^*N*^ *P′*^*i*^_AB_*P′*^*i*^_AC_	∑_*i*=1_^*N*^ *P′*^*i*^_*AC*_ (1*−P′*^*i*^_*AB*_)
Incorrect (1−*P*_AC_)	∑_*i*=1_^*N*^ *P′*^*i*^_AB_ (1*−P′*^*i*^_AC_)	∑_*i*=1_^*N*^ (1*−P′*^*i*^_*AB*_)(1*−P′*^*i*^_*AC*_)

The dependent model replaces the probability of correctly recalling B when cued by A (across all events; *P*_AB_) with *P′*^*i*^_AB_=*E*^*i*^_AB_(*P*_AB_*−P*_G_/*c*)*+P*_G_/*c,* where the ‘episodic factor' *E*^*i*^_AB_ reflects performance on event *i* relative to other events (based on retrievals other than B and C cued by A), *P*_G_ is the probability of guessing and *c*=6 is the number of choices in a test trial. *P*_AC_ is replaced similarly.
