# Supplementary material for: Evidence for holistic episodic recollection via hippocampal pattern completion
Source: Nat Commun. 2015 Jul 2;6:7462. doi: 10.1038/ncomms8462 (PMC4506995; doi:10.1038/ncomms8462)
Supplement: Supplementary Information — Supplementary Figure 1, Supplementary Tables 1-4 and Supplementary Notes 1-5 [file ncomms8462-s1.pdf]

Supplementary Information

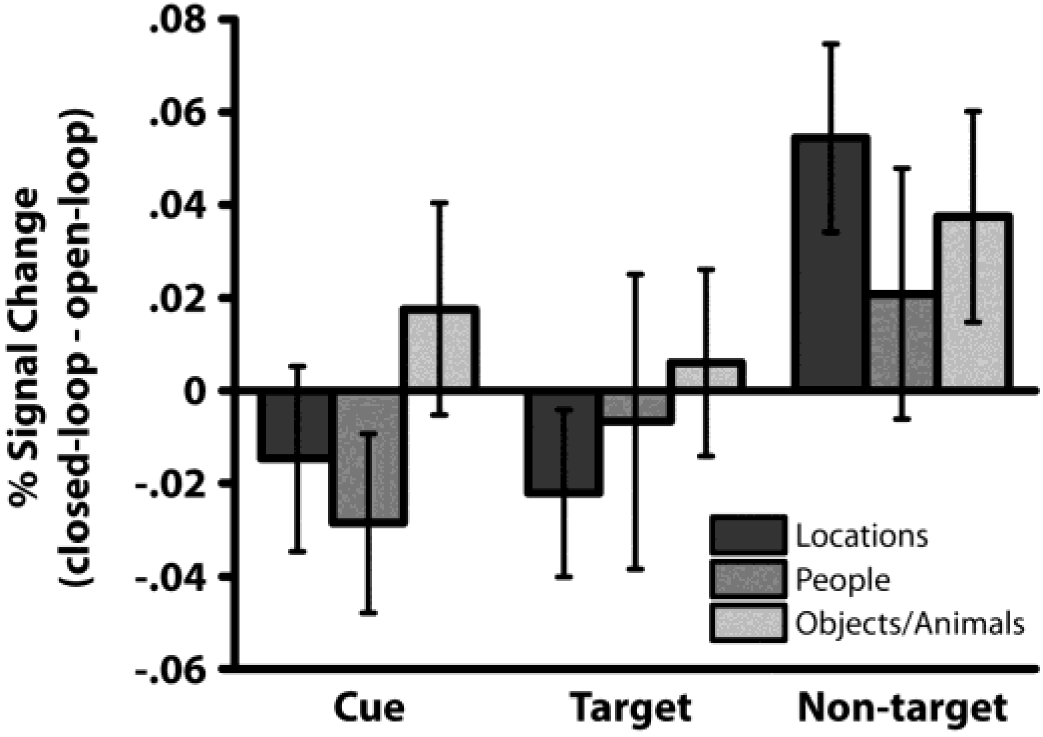

Supplementary Figure 1. Non-target reinstatement across neocortical regions. Mean % signal change for the cue, target and non-target (closed-loop vs. open-loop events) across the three element-specific neocortical regions of interest (locations, people and objects/animals). Error bars +/- 1 standard error.

Supplementary Table 1. Clusters and their peaks showing element specific cortical responses for locations, people and objects/animals at encoding (p<.05 FWE corrected). L = left hemisphere; R = right hemisphere. Regions in **bold** shown in Fig 4.

Region	Voxels	MNI coordinates			Z Score
		x	y	z	
Locations					
**L parahippocampal gyrus**	**67**	**-30**	**-40**	**-8**	**7.26**
R medial parietal	87	+18	-55	+19	6.27
L lateral parietal	48	-39	-79	+28	5.91
L medial parietal	27	-15	-55	+16	5.66
R parahippocampal gyrus	13	+30	-37	-11	5.43
People					
**Medial parietal**	**46**	**+3**	**-58**	**+31**	**5.72**
L lateral anterior temporal	11	-63	+2	-17	5.55
Medial prefrontal cortex	18	0	+59	-14	5.27
Medial prefrontal cortex	17	+3	+44	-20	5.13
L posterior lateral occipital	5	-18	-94	-2	4.86
Objects/Animals					
**L lateral occipital cortex**	**16**	**-54**	**-64**	**+1**	**5.23**
L lateral parietal cortex	12	-60	-31	+37	5.07

Supplementary Table 2. Clusters and their peaks for the subsequent memory analysis of the encoding phase (p<.05 FWE SVC). L = left hemisphere; R = right hemisphere. Regions in **bold** shown in Fig 4.

		MNI coordinates			
Region	Voxels	x	y	z	Z Score
**R hippocampus**	**79**	**+36**	**-13**	**-14**	**3.61**
L hippocampus	24	-27	-7	-26	3.50

Supplementary Table 3. Clusters and their peaks showing element specific cortical responses for locations, people and objects/animals at retrieval ($p<.05$ FWE corrected). L = left hemisphere; R = right hemisphere. Regions in **bold** shown in Fig 5. Note, the frontal region for objects/animals was not seen at encoding (unlike the peaks for locations and people) and was not predicted. Given this, we focused on the next peak in the left lateral parietal cortex (also seen at encoding; -51, -46, +43) for all subsequent analyses.

Region	Voxels	MNI coordinates			Z Score
		x	y	z	
Locations					
**L parahippocampal gyrus**	**390**	**-30**	**-34**	**-14**	**>7.84**
R parahippocampal gyrus	104	+30	-31	-14	>7.84
L lateral parietal	128	-39	-82	+31	7.46
R cuneus	138	+12	-49	+7	7.14
R lateral parietal	32	+48	-73	+25	6.17
L lateral occipital	58	-54	-52	-5	5.74
People					
**Medial prefrontal cortex**	**217**	**0**	**+41**	**-20**	**>7.84**
Medial parietal	233	+6	-55	+25	>7.84
R lateral anterior temporal	28	+63	-1	-20	6.53
L posterior occipital	115	-18	-88	-14	6.52
R posterior occipital	15	+18	-91	-2	5.24
Objects/Animals					
L inferior frontal gyrus	84	-48	+41	+13	6.17
**L lateral parietal**	**20**	**-51**	**-46**	**+43**	**5.63**
L lateral occipital cortex	1	-57	-58	-5	4.77

Supplementary Table 4. Clusters and their peaks showing regions correlating with the difference between non-target closed-loop vs open-loop trials at retrieval ($p<.05$ FWE SVC). L = left hemisphere; R = right hemisphere. Regions in **bold** shown in Fig 5.

Region	Voxels	MNI coordinates			Z Score
		x	y	z	
**R hippocampus**	**103**	**+33**	**-22**	**-8**	**4.84**
L hippocampus	143	-30	-28	-11	4.66

Supplementary Note 1

Mean performance across participants was relatively high (64%). We first analysed performance across Cue-type (locations, people and objects/animals) and closed-loop vs open-loop events in a 3x2 within-subject ANOVA. This revealed a main effect of closed-loop vs open-loop events, $F(1, 25) = 12.31$, $p<.01$, $\eta_P^2=.33$, with better performance for closed-loop (68%) than open-loop (61%) events. Neither the main effect of Cue-type nor the interaction approached significance, F's<2.4, p's>.10, η_P^2's<.09. A 3x2 ANOVA for Target-type revealed a similar main effect of closed-loop vs open-loop events, $F(1, 25) = 12.31$, $p<.01$, $\eta_P^2=.33$. A main effect of Target-type was also present, $F(1.7, 43.5) = 11.11$, $p<.001$, $\eta_P^2=.31$, and an interaction, $F(2.0, 49.3) = 3.31$, $p<.05$, $\eta_P^2=.12$. The main effect was characterised by better performance when retrieving the location relative to people and objects, a difference that was more pronounced in the open-loop than closed-loop condition. This greater variability in memory performance across elements in the open-loop condition accords with our previous work [27] and is consistent with the presence of pattern completion in the closed-loop but not open-loop condition. The proportions of trials for each confidence value (1-4) were as follows: 0.34, 0.11, 0.13, 0.42. For closed-loop events it was 0.34, 0.10, 0.12, 0.44 and for open-loop events it was 0.34, 0.12, 0.14, 0.40. Thus, participants predominantly rated their confidence as either low (1) or high (4), with a small increase in their use of value 4 in the closed-loop than open-loop condition.

Supplementary Note 2

Before comparing the dependency in the data with the independent and dependent models, we first assessed raw dependency for the data across Analysis-type (AbAc vs BaCa) and Element-type (locations vs people). This 2x2x2 (Analysis-type x Element-type x closed-loop vs open-loop) within-subjects ANOVA revealed main effects of closed-loop vs open-loop, $F(1, 25) = 11.52$, $p<.01$, $\eta_P^2=.32$, and Element-type, $F(1, 25) = 9.92$, $p<.01$, $\eta_P^2=.28$. This latter effect was characterised by greater dependency in the data for locations relative to people. Given the raw dependency measure scales with accuracy (i.e., higher accuracy = higher dependency), these two main effects are perhaps related to better performance in the closed-loop relative to open-loop condition and the better performance for locations relative to people. No further main effects or interactions approached significance, *F*'s<.59, p's>.45. We collapsed across Analysis-type and Element-type prior to comparing dependency in the data to the two models, as presented in the main text (Fig 3).

Supplementary Note 3

We entered the neocortical retrieval data into a 3x3x2 (region x element-status x closed-loop vs open-loop) within-subject ANOVA, where element-status refers to whether the element was a cue, target or non-target. Importantly, we saw an element-status x closed-loop vs open-loop interaction, $F(1.8, 45.9) = 7.50$, $p<.01$, $\eta_P^2=.23$ (the pattern for this interaction is described in the main text). Importantly, this effect did not significantly differ across the three regions, shown by a lack of a three-way interaction, $F(3.5, 86.3) = .49$, $p=.71$, $\eta_P^2=.02$. Thus, the difference between closed-loop and open-loop events for non-target trials was consistent across regions. This is important as is suggests that the lateral parietal region associated with both objects and animals did not differ from the other two regions. Thus, despite this region coding for two elements it showed the same pattern as those associated with a single element.

Further main effects of region, $F(1.9, 46.7) = 46.72$, $p<.001$, $\eta_P^2=.65$, and element-status, $F(1.7, 41.9) = 105.68$, $p<.001$, $\eta_P^2=.81$, were seen. The former relates to differences across regions relative to the ITI period, whilst the regions were defined based on the latter (i.e., cued/targets vs. non-targets). Finally, a region x element-status interaction was also seen, $F(2.5, 61.6) = 7.00$, $p<.001$, $\eta_P^2=.22$.

Supplementary Note 4

We conducted an analysis to rule out that this non-target reinstatement effect was being driven by a specific subset of retrieval trials in which the object/animal is the cue. To test whether these trials were driving our non-target reinstatement effect (which is already unlikely because the effect does not differ across the three neocortical regions), we conducted an analysis directly comparing non-target reinstatement for people or places between trials in which a direct association between cue and non-target was present only in closed-loop events and trials in which the direct association was present in both conditions. A 2x2x2 ANOVA (closed-loop vs. open-loop x Analysis x Region) was conducted, where Analysis refers to whether the trials were balanced for direct cue-to-non-target associations and Region refers to the 2 regions corresponding to the non-target type (parahippocampal gyrus and medial prefrontal cortex). This revealed a significant main effect of closed-loop vs. open-loop, $F(1, 25) = 5.61$, $p<.05$, $\eta_P^2=.18$, confirming the main result of non-target reinstatement for closed-loop events. Importantly, no further main effects or interactions were seen, F's<2.7, p's>.11, $\eta_P^2<.10$. Thus, the non-target reinstatement effect was not affected by the presence or absence of a direct cue-to-non-target association.

Supplementary Note 5

The data from the post-scan debrief was condensed into 6 variables: (1) proportion of pairs participants remembered seeing in the encoding or retrieval phase, (2) proportion of pairs participants remembered seeing in the encoding phase, (3) how vividly they imagined the two items interacting at encoding (scale 1-4), (4) proportion of pairs where they (correctly) brought any other associated element to mind during encoding, (5) proportion of pairs participants remembered seeing in the retrieval phase and (6) proportion of pairs where they (correctly) brought any other associated element to mind during retrieval. Each of these variables was calculated for the closed-loop and open-loop events separately.

We first compared the closed-loop and open-loop conditions for each of the 6 variables. All contrasts showed a significant difference (t's>2.36, p's<.05), suggesting that closed-loop events were (1) better remembered, in accord with actual memory performance measures, (2) imagined more vividly and (3) more often associated with the retrieval of non-target elements. We next performed a linear regression analysis correlating the difference between closed-loop and open-loop conditions for each of the 6 variables with behavioural dependency (closed-loop – open-loop, relative to their respective independent model baselines) across participants. The only variable that showed a significant correlation with behavioural dependency was the proportion of pairs where they (correctly) brought another element from the same event to mind at retrieval (R^2=.19, p<.05; remaining regressions: R^2's<.03, p's>.44). Thus, the extent to which participants showed behavioural dependency showed a close correspondence with the extent to which they brought the non-target event element to mind at retrieval.
